# Oncogenic USP22 supports gastric cancer growth and metastasis by activating c-Myc/NAMPT/SIRT1-dependent FOXO1 and YAP signaling

**DOI:** 10.18632/aging.102410

**Published:** 2019-11-04

**Authors:** Hongxia Liu, Ningning Liu, Yali Zhao, Xiaoshan Zhu, Changsong Wang, Qinqin Liu, Chunfang Gao, Xusheng Zhao, Juntang Li

**Affiliations:** 1 Jujube Scientific Research and Applied Center, Life Science College, Luoyang Normal University, Luoyang, Henan 471934, China; 2 Centre of Inflammation and Cancer Research, 150th Central Hospital of PLA, Luoyang, Henan 471031, China; 3 Department of Pathology, 150th Central Hospital of PLA, Luoyang, Henan 471031, China; 4 State Key Laboratory of Cancer Biology, Department of Immunology, Fourth Military Medical University, Xi’an, Shaanxi 710032, China; 5 State Key Laboratory of Cancer Biology, Department of Biochemistry and Molecular Biology, Fourth Military Medical University, Xi’an, Shaanxi 710032, China

**Keywords:** ubiquitin-specific peptidase 22, gastric cancer, proliferation, invasion, apoptosis

## Abstract

In this study, we investigated the role of ubiquitin-specific protease 22 (USP22) in the growth and progression of gastric cancer (GC). USP22 mRNA and protein levels were significantly higher in GC tissue samples and GC cell lines than in adjacent noncancerous tissue samples and a normal gastric mucosal epithelial cell line (GES1), respectively. USP22 knockdown significantly decreased *in vitro* survival, proliferation, migration, and invasiveness of GC cells compared with the controls. Western blot analysis of control and USP22-silenced GC cells showed that USP22 modulates the c-Myc/NAMPT/SIRT1-dependent FOXO1 and YAP signaling pathways. Subcutanenous injection of USP22-silenced GC cells into SCID mice generated significantly smaller xenograft tumors than did control cells. Moreover, USP22-silenced GC cells showed less lung metastasis than the controls following tail vein injection in SCID mice. In addition, high USP22 expression correlated positively with tumor size, advanced stage and metastasis, and correlated negatively with tumor differentiation and prognosis in GC patients. These results show that USP22 regulates growth and progression of GC via the c-Myc/NAMPT/SIRT1-dependent FOXO1 and YAP signaling pathways.

## INTRODUCTION

Gastric cancer (GC) is the second most common malignancy and the third leading cause of cancer-related deaths in China [1]. A large number of patients are diagnosed with advanced GC (stages III and IV), which is associated with poor prognosis [2]. The five-year survival rates of GC patients is below 30% despite great advances in GC therapy [3]. Hence, novel therapeutic strategies are urgently needed to improve survival rates of GC patients.

Ubiquitin-specific protease 22 (USP22) is a deubiquitinating enzyme and an integral component of the 11-gene polycomb/cancer stem cell signature; it is an important subunit of the human Spt-Ada-Gcn5 acetyltransferase (hSAGA) complex that is required for transcriptional regulation and cell cycle progression [4, 5]. USP22 regulates transcription of genes related to epigenetic alterations and cancer progression by deubiquitination of H2A or H2B histones [4, 5]. USP22 is highly expressed in several cancers [6–8], which considerably contributes to tumor recurrence, distant metastasis, therapeutic failure, and poor prognosis [9]. Downregulation of USP22 reduces *in vitro* cancer cell proliferation, survival, migration, and invasion, and decreases *in vivo* tumor growth and metastasis [6, 10–13]. He *et al* reported increased co-expression of USP22 and c-Myc in GC tissues [7]. USP22 depletion in the lung cancer cells decreases the transcriptional ability of Myc [4]. Sirtuin1 (SIRT1) expression is c-Myc-dependent [14], and its activity is increased in a majority of cancers including GC [15, 16]. Moreover, SIRT1 activity requires c-Myc-dependent induction of the nicotinamide phosphoribosyltransferase (NAMPT) [17]. SIRT1 is an NAD^+^-dependent deacetylase that regulates aging, DNA repair, cell cycle, metabolism, and cell survival [14, 17]. SIRT1 promotes breast cancer progression by targeting many transcription factors, such as p53 [18], forkhead transcription factors of the O class 1 (FOXO1) [19], and Yes-associated protein (YAP) [20]. Previous reports suggest that FOXO1 enhances apoptosis of prostate cancer cells [21] and cell cycle arrest [22]. FOXO1 is downregulated in several GC tissue samples, and constitutive phosphorylation of the FOXO1A correlates with better prognosis of GC [23]. YAP is a downstream effector of the Hippo pathway and acts as an oncogene in multiple cancers including GC [24]. YAP increases GC cell motility by increasing the expression of matrix metalloproteinases, MMP-2 and MMP-9 [24].

The biological functions of USP22 and the underlying signaling pathways involved in GC progression are not clear. Therefore, we investigated the status of USP22 expression in GC tissues and cell lines and its biological role in GC progression. Our findings demonstrate that USP22 is a key player in GC progression and a potential target for GC therapy.

## RESULTS

### USP22 is upregulated in GC tissues and cell lines

**Figure 1:**
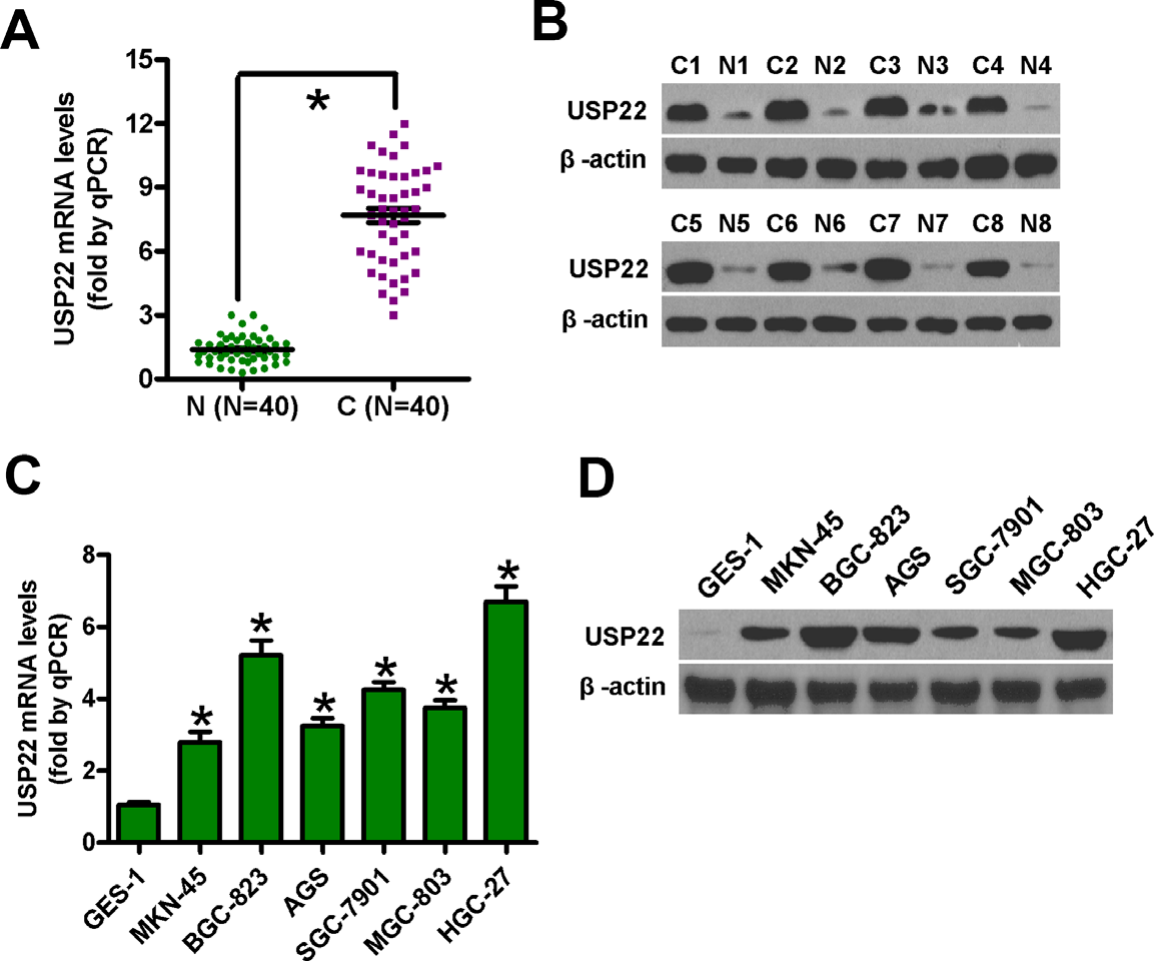
**USP22 mRNA and protein levels in GC tissues and cell lines.** (**A**) Histogram plots show USP22 mRNA levels based on qRT-PCR analysis in 40 pairs of GC (C) and adjacent noncancerous (N) tissues. GAPDH was used as an endogenous control. (**B**) Representative images show western blot analysis of USP22 protein levels in eight pairs of GC (**C**) and adjacent noncancerous tissues (N). β-actin was used as endogenous control. (**C**–**D**) The levels of USP22 mRNA (**C**) and protein (**D**) in GC cell lines and normal gastric mucosal epithelial cells are shown in the histogram plots and representative images, respectively. GAPDH and β-actin were used as the controls in the qRT-PCR and western blot analysis, respectively. Data are expressed as mean ± SD of three replicates. *p < 0.05 is considered statistically significant.

We analyzed the levels of USP22 mRNA and protein expression in GC tissues and cell lines relative to the corresponding controls. Quantitative RT-PCR (n=40 samples each) and western blot (n=8 samples each) analyses showed that the levels of USP22 mRNA and protein expression were significantly higher in the GC tissues compared with the corresponding adjacent noncancerous tissues (Figure 1A, 1B). Moreover, the levels of USP22 mRNA and protein expression were significantly higher in the six GC cell lines (BGC-823, MGC-803, SGC-7901, MKN-45, AGS, and HGC-27) compared with the normal gastric mucosal epithelial cell line, GES-1 (Figure 1C, 1D). These data suggest that USP22 is overexpressed in GC tissues and cell lines compared with the corresponding controls. Since the MGC-823 and HGC-27 cell lines showed the highest levels of USP22 mRNA and protein expression among the six GC cell lines, both cell lines were selected for further studies.

### USP22 knockdown inhibits GC cell proliferation *in vitro*

**Figure 2:**
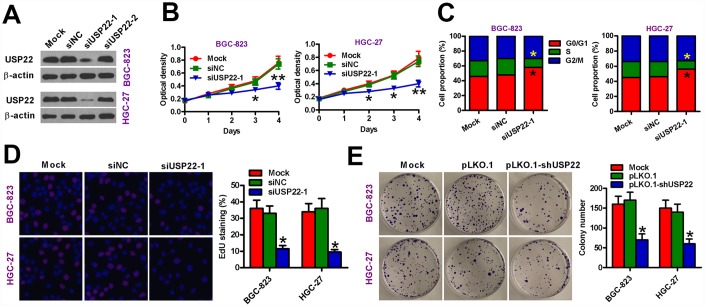
**USP22 silencing inhibits *in vitro* GC cell proliferation.** (**A**) Representative images show USP22 expression in BGC-823 and HGC-27 cells that were transfected with 100 nM siNC, siUSP22-1, or siUSP22-2 cultured for 24 h. Untransfected (mock) GC cells were used as controls. β-actin was used as an endogenous control. (**B**) MTT assay results show viability of siNC or siUSP22-1 transfected GC cells at days 1, 2, 3, and 4. (**C**) Flow cytometry analysis shows the effects of USP22 knockdown in GC cells. The percentage of G1, S-, and G2M cells in siNC or siUSP22-1 transfected GC cells are shown in the histogram plots. (**D**) EdU assay results show cell proliferation status of siNC or siUSP22-1 transfected GC cells. The plots show the percentage of EdU-positive cells in various samples. Magnification: 100×. (**E**) Histograms (right) show the total number of colonies in BGC-823 and HGC-27 cells that are uninfected (Mock) or infected with lentiviruses carrying pLKO.1 or pLKO.1-shUSP22 vectors. Representative images of colony formation assay are also shown. Note: Data are expressed as mean ± SD of three replicates; *p < 0.05; **p < 0.01 compared with mock or siNC group in (**B**–**D**); *p < 0.05 compared with the mock or pLKO.1 group in (**E**).

To silence USP22, we transfected BGC-823 and HGC-27 GC cell lines with siRNAs against USP22 and observed that siUSP22-1 significantly downregulated USP22 compared with the GC cell lines transfected with the control siRNA, siNC (Figure 2A). MTT assay results showed that USP22 silencing significantly reduced the viability of siUSP22-1-transfected BGC-823 and HGC-27 cells compared with the mock and siNC-transfected GC cells (Figure 2B). Flow cytometry analysis showed that USP22 silencing resulted in G1-cell cycle arrest in the siUSP22-1-transfected BGC-823 and HGC-27 cells based on significantly higher percentage of G1-phase cells than S and G2M phase cells compared with the mock and siNC-transfected GC cells (Figure 2C). Moreover, EdU assay results showed that USP22 knockdown significantly reduced proliferation of BGC-823 and HGC-27 cells compared with the controls (Figure 2D). We further studied the effect of USP22 silencing on GC cell proliferation by infecting BGC-823 and HGC-27 cells with lentiviruses containing the pLKO.1-shUSP22 or the control pLKO.1 vectors. The inferring efficiency of pLKO.1-shUSP22 in GC cells sees the Supplementary Figure 1. The pLKO.1-shUSP22 group BGC-823 and HGC-27 GC cells with low USP22 expression formed significantly fewer and smaller colonies than the pLKO.1 group GC cells (Figure 2E). These data suggest that USP22 is required for *in vitro* proliferation and survival of GC cell lines.

### USP22 depletion promotes *in vitro* GC cell apoptosis

**Figure 3:**
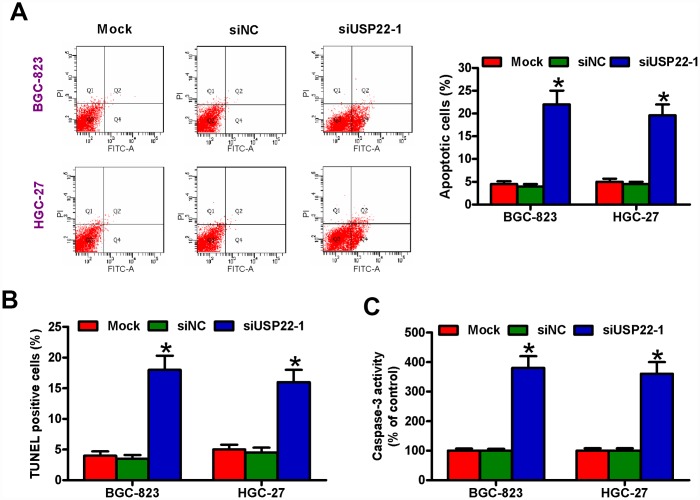
**USP22 knockdown induces *in vitro* GC cell apoptosis.** The results of (**A**) Annexin V/PI staining, (**B**) TUNEL, and (**C**) caspase-3 activity assays in BGC-823 and HGC-27 cells that are untransfected (Mock) or transfected with 100 nM siNC or siUSP22-1are shown. Note: Data are expressed as mean ± SD of three replicates; *p < 0.05 compared with the mock or siNC group.

Next, we investigated the role of USP22 in the survival of GC cells. Analysis of AnnexinV/Propidium Iodide stained cells by flow cytometry showed significantly increased apoptosis in the USP22-silenced GC cells compared with the mock- and siNC-transfected GC cells (Figure 3A). USP22 silencing increased the percentage of TUNEL-positive BGC-823 and HGC-27 cells compared with the controls (Figure 3B). Moreover, USP22-silenced BGC-823 and HGC-27 cells showed significantly higher caspase-3 activity compared with the controls (Figure 3C). These data demonstrate that USP22 expression is required for the survival of GC cells *in vitro*.

### USP22 silencing suppresses *in vitro* GC cell migration and invasiveness

**Figure 4:**
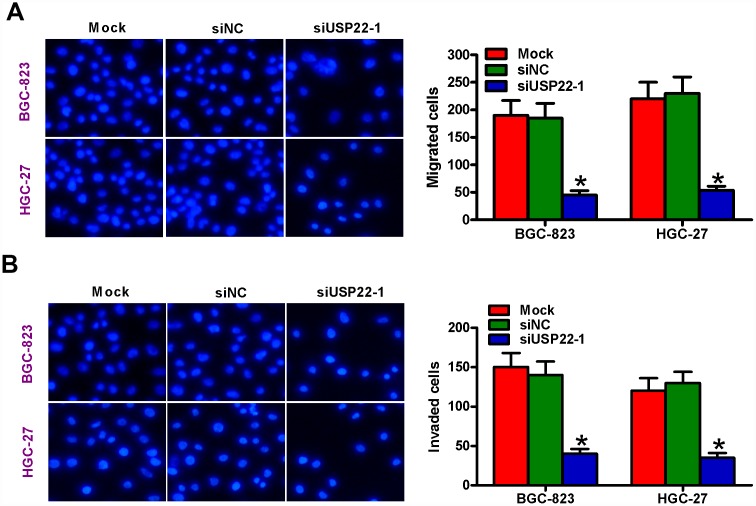
**USP22 silencing suppresses *in vitro* GC cell migration and invasion.** (**A**–**B**) Transwell assay results show total number of (**A**) migratory and (**B**) invasive BGC-823 and HGC-27 cells that are untransfected (Mock) or transfected with 100 nM siNC or siUSP22-1 at 48 h after transfection. Note: Magnification: 200×; Data are expressed as mean ± SD of three replicates. *p < 0.05 compared with mock or siNC group.

We then performed Transwell migration and invasion assays to determine the role of USP22 in GC cell motility. Migration and invasiveness of USP22-silenced BGC-823 and HGC-27 cells was significantly reduced compared with the mock- and siNC-transfected BGC-823 and HGC-27 cells (Figure 4A, 4B). These results suggest that USP22 promotes *in vitro* GC cell migration and invasion.

### USP22 promotes *in vitro* GC progression by modulating c-Myc/NAMPT/SIRT1- dependent FOXO1 and YAP signaling pathways

**Figure 5:**
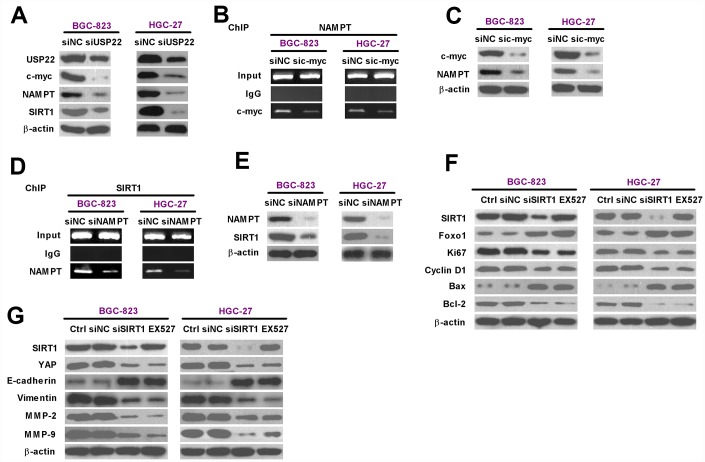
**USP22 promotes *in vitro* GC progression via c-Myc/NAMPT/ SIRT1-dependent FOXO1 and YAP signaling pathways.** (**A**) Representative images show the levels of USP22, c-Myc, NAMPT, and SIRT1 proteins in BGC-823 and HGC-27 cells transfected with 100 nM siNC or siUSP22-1. The cells were analyzed at 24 h after transfection. β-actin was used as an endogenous control. (**B**) ChIP assay analysis shows binding efficiency of c-Myc in the promoter region of NAMPT in BGC-823 and HGC-27 cells that were transfected with 100 nM sic-Myc or siNC. (**C**) Representative images show the levels of c-Myc and NAMPT proteins in BGC-823 and HGC-27 cells that were transfected with 100 nM sic-Myc or siNC. β-actin was used as endogenous control. (**D**) ChIP assay analysis shows binding efficiency of NAMPT in the promoter region of SIRT1 in BGC-823 and HGC-27 cells that were transfected with 100 nM siNAMPT or siNC. (**E**) Representative images show the levels of NAMPT and SIRT1 protein in BGC-823 and HGC-27 cells were transfected with 100 nM siNAMPT or siNC. β-actin was used as endogenous control. (**F**–**G**) Representative images show the levels of (**F**) Sirt1, FOXO1, Ki67, Cyclin D1, Bax, and Bcl-2, and (**G**) Sirt1, YAP, MMP-2, and MMP-9 proteins in BGC-823 and HGC-27 cells transfected with 100 nM siSirt1 or siNC, or cells treated with 100 μM EX527, a Sirt1 inhibitor. β-actin was used as an endogenous control.

We then investigated the mechanism by which USP22 drives GC progression by analyzing the status of various signaling pathways in the USP22-silenced and control GC cells. Western blot analysis showed that c-Myc, NAMPT, and SIRT1 protein levels were significantly reduced in the USP22-silenced BGC-823 and HGC-27 cells compared with the controls (Figure 5A). Previous studies have shown that cMYC binds to two conserved E-boxes in the promoter region of the *NAMPT* gene [17, 25]. Therefore, we performed ChIP analysis and observed that c-Myc knockdown significantly reduced the occupancy of c-Myc in the promoter region of the *NAMPT* gene (Figure 5B, 5C). Moreover, western blot analysis showed that the expression of NAMPT protein was significantly reduced in the c-Myc-silenced BGC-823 and HGC-27 cells compared with the controls (Figure 5C). Previous reports suggest that NAMPT is a positive regulator of SIRT1 transcription [17]. Therefore, we performed ChIP analysis and observed that NAMPT knockdown reduced the levels of NAMPT bound in the *SIRT1* promoter compared with the controls (Figure 5D and 5E). Furthermore, SIRT1 expression was significantly reduced by silencing NAMPT (Figure 5E).

We further analyzed the role of USP22-dependent c-Myc/NAMPT/SIRT1 signaling in GC progression. SIRT1 silencing by siSIRT1 transfection of BGC-823 and HGC-27 cells or treatment of BGC-823 and HGC-27 cells with the SIRT1 inhibitor, EX527, showed significantly higher levels of FOXO1 and pro-apoptotic protein, Bax; concurrently, the levels of pro-proliferative proteins, Ki67 and Cyclin D1, and the anti-apoptotic protein, Bcl-2, were significantly lower (Figure 5F). Moreover, SIRT1 knockdown or SIRT1 activity inhibition by EX527 reduced the levels of YAP, vimentin (mesenchymal marker), MMP-2 and MMP-9, and increased the levels of E-cadherin expression (epithelial marker) in the BGC-823 and HGC-27 cells (Figure 5G). These data suggest that USP22 promotes GC progression by modulating c-Myc/NAMPT/ SIRT1-dependent FOXO1 and YAP signaling.

### USP22 depletion attenuates *in vivo* GC growth and metastasis

**Figure 6:**
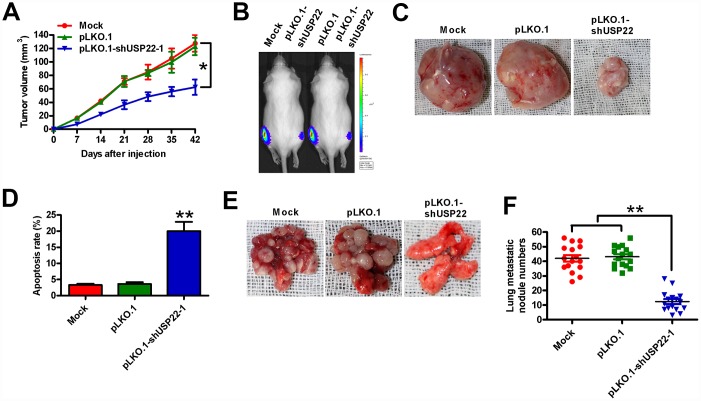
**USP22 depletion reduces *in vivo* GC growth and metastasis.** Six-week-old male SCID mice were subcutaneously injected with stably-expressed pLKO.1-shUSP22-1 or pLKO.1 BGC-823-luc cells into the hind flanks or through the tail vein. (**A**) Histogram shows tumor volume at weeks 1, 2, 3 and 4 after subcutaneous injection of SCID mice (n=5 each) with BGC-823-luc cells stably-expressing pLKO.1-shUSP22-1 or pLKO.1 BGC-823-luc cells into the hind flanks at 1, 2, 3 and 4 weeks. (**B**) Analysis of tumor growth progression by *in vivo* luciferase imaging of the xenografts at day 21 after subcutaneous injection of SCID mice (n=5 each) with stably-expressed pLKO.1-shUSP22-1 or pLKO.1 BGC-823-luc cells. (**C**) Representative photographs of xenograft tumors at day 42 from SCID mice subcutaneously injected with stably-expressed pLKO.1-shUSP22-1 or pLKO.1 BGC-823-luc cells. (**D**) TUNEL assay results show the total number of apoptotic cells in the xenograft tumor sections derived at day 42 from from SCID mice subcutaneously injected with stably-expressed pLKO.1-shUSP22-1 or pLKO.1 BGC-823-luc cells. (**E**) Representative images and (**F**) Total number of s metastatic nodules in the lungs at day 28 from SCID mice that were injected with stably-expressed pLKO.1-shUSP22-1 or pLKO.1 BGC-823-luc cells. Note: Data are expressed as mean ± SD of three replicates; *p < 0.05, **p < 0.01 compared with the mock or pLKO.1 group.

We then investigated the *in vivo* role of USP22 in tumor growth and metastasis by establishing tumor xenograft and metastasis models in SCID mice. We established tumor xenografts by subcutaneously injecting USP22 knockdown or control BGC-823-luc cells into 6-week-old male SCID mice (*n* = 5 each). We observed visible tumors within a week in both groups, but there was a significant time-dependent reduction in the growth of tumors in the USP22 knockdown group compared with the control group (Figure 6A). At days 21 and 42, the sizes of tumors were significantly smaller in the USP22 knockdown group compared with the tumors in the control group (Figure 6B and 6C). The mice were sacrificed 6 weeks after tumor cell implantation. USP22 reduction at its mRNA and protein levels was observed in the tumor tissues from the mice with USP22-silenced BGC-823-luc cells (Supplementary Figure 2A and 2B). TUNEL assay analysis of xenograft tumor sections showed significantly higher number of apoptotic cells in the USP22 knockdown group compared with the control group (Figure 6D). For *in vivo* metastasis assays, we injected the USP22 knock down or control BGC-823-luc cells into the tail veins of the SCID mice (*n* = 5 each). As shown in Figure 6E and 6F, SCID mice that received USP22 knockdown BGC-823-luc cells developed significantly fewer lung metastatic nodules compared with the control groups. These data demonstrate that USP22 promotes *in vivo* GC growth and metastasis.

### Upregulation of USP22 in GC tissues is positively associated with the advanced histological grade, metastasis, and poor prognosis

**Figure 7:**
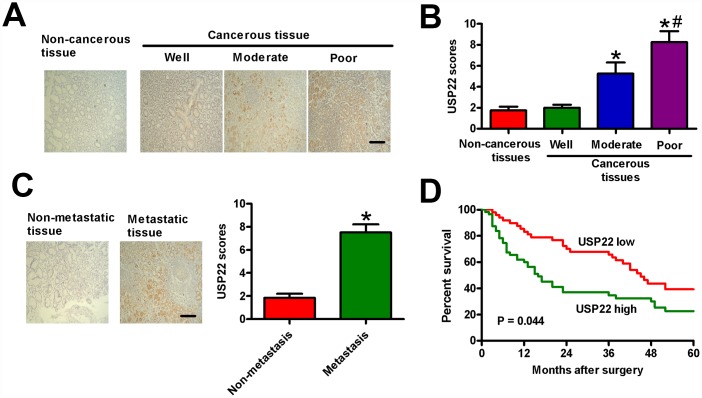
**USP22 overexpression positively correlates with advanced histological grade, metastasis, and poor prognosis in GC patients.** (**A**) Representative images of IHC staining of USP22 and (**B**) USP22 expression scores in the sections of non-cancerous and GC tissues at various stages of differentiation. Scale bar: 10 μm. (**C**) USP22 expression scores in metastatic and non-metastatic GC tissue samples (n = 40). (**D**) Relationship between levels of USP22 expression based on IHC and the five-year overall survival (OS) rates after surgery in GC patients with high-USP22 (n = 107) and low-USP22 (n = 79) expression. Note: Data are expressed as mean ± SD of three replicates; *p < 0.05 compared with non-cancerous tissues or well-differentiated tissues in (**B**); *p < 0.05 compared with non-metastatic GC tissues in (**C**); ^#^*p* < 0.05 compared with moderate-differentiated tissues in (**B**).

We then performed IHC assays in 186 paraffin-embedded human GC tissue samples to understand the relationship between USP22 expression and various clinicopathological parameters in GC. The 186 samples were categorized into high-USP22 (n = 107) and low-USP22 (n = 79) expression groups. High USP22 expression correlated with large tumor size, tumor differentiation, tumor infiltration, local lymph node metastasis, distant metastasis, and TNM stage (Table 1). The degree of tumor differentiation negatively correlated with the level of USP22 protein expression in the GC tissue samples (Figure 7A and 7B). The metastatic GC tissue samples showed significantly higher USP22 protein expression than the non-metastatic GC tissue samples (Figure 7C). Kaplan Meier survival curve analysis showed that five-year overall survival (OS) in patients with low USP22 protein expression was significantly higher than in patients with high USP22 protein expression (Figure 7D). These results suggest that high USP22 expression in GC tissues is positively correlated with advanced histological tumor grade, metastasis, and poor prognosis.

**Table 1:** The correlation of USP22 expression with clinicopathological features of gastric cancer.

**Variables**	**Clinicopathological parameters**	**Case No. (n = 186)**	**USP22 expression**		**P value**
			**High (n = 107)**	**Low (n = 79)**	
Gender	Male	119	69	50	0.788
	Female	67	38	29	
Age (years)	< 60	85	43	42	0.079
	≥ 60	101	64	37	
Size (cm)	< 5	37	29	8	0.005*
	≥ 5	149	78	71	
Differentiation	Well	11	6	5	0.017*
	Moderate	49	32	17	
	Poor	126	69	57	
Tumor infiltration	T1	29	24	5	< 0.001*
	T2	21	18	3	
	T3	15	7	8	
	T4	121	58	63	
Local lymph node metastasis	N0	58	37	21	0.022*
	N1	35	22	13	
	N2	36	13	23	
	N3	57	35	22	
Distant metastasis	M0	166	90	76	0.013*
	M1	20	17	3	
TNM stage	I	37	31	6	
	II	35	15	20	
	III	94	49	45	< 0.001*
	IV	20	12	8	

*P < 0.05.

## DISCUSSION

Our study demonstrates that USP22 plays a significant role in GC progression. We demonstrate that USP22 mRNA and protein levels are significantly upregulated in the GC tissues and cell lines. USP22 knockdown decreases *in vitro* proliferation, migration and invasiveness of GC cells. Moreover, USP22 knockdown induces apoptosis in GC cells. USP22 promotes GC progression by modulating FOXO1 or YAP signaling pathways via c-Myc/NAMPT/SIRT1. *In vivo* experiments reveal that USP22 knockdown in GC cells significantly reduces xenograft tumor growth and lung metastasis in SCID mice. Moreover, high USP22 expression in GC tissues positively correlates with tumor size, local lymph node metastasis, distant metastasis, and histological grades, and negatively correlates with GC differentiation and prognosis.

Several studies have shown that USP22 plays a key role in tumorigenesis and is a potential diagnostic and prognostic marker of human cancers [26, 27]. In this study, we demonstrate that USP22 is overexpressed in GC tissues compared with the matched adjacent non-cancerous tissues. High levels of USP22 protein in the GC tissues positively correlate with several clinicopathological characteristics, suggesting an oncogenic role for USP22 in GC. Previous studies have shown that USP22 plays an oncogenic role by activating the polycomb genes, which are mechanistically linked to the pathogenesis of several solid tumors [9]. Moreover, Kaplan–Meier survival curve analysis shows that five year OS is significantly reduced in GC patients with high USP22 expression than GC patients with low USP22 expression. This result suggests that USP22 is a potential diagnostic and a prognostic marker for GC patients.

Previous studies show that USP22 promotes proliferation and survival of anaplastic thyroid [6], hepatic [13], colorectal [28], and bladder [29] cancer cells. USP22 silencing promotes G1-phase cell cycle arrest, thereby suggesting that USP22 promotes G1-to-S cell cycle transition [13, 28, 29]. Previous studies have shown that USP22 expression positively correlates with c-Myc and BMI expression in GC tissues [7, 8]. USP22 is required for several functions of c-Myc including malignant transformation of normal cells [4]. c-Myc plays a central role in controlling cell cycle progression of tumor cells [4, 5]. USP22 increases the stability and tumorigenic activity of c-Myc in breast cancer cells [30]. USP22 also promotes the proliferation of HepG2 and HeLa cells via the c-Myc/cyclin D2 signaling pathway [13, 31]. Moreover, USP22 promotes cell cycle progression by regulating BMI-mediated INK4a/ARF and Akt signaling pathways [12, 32–34]. USP22 knockdown in anaplastic thyroid carcinoma cells induces mitochondria-dependent apoptotic pathway by upregulating levels of apoptotic protein, Bax and Bid, and activating caspase-3 [6]. Our study also shows that USP22 silencing in GC cells decreases cell proliferation and induces cell cycle arrest and apoptosis *in vitro*, and suppresses tumor growth and metastasis *in vivo*. Overall, these results suggest that USP22 plays an essential role in GC growth and progression.

USP22 is positively associated with metastasis of several cancers [6, 26, 27]. USP22 knockdown reduces the invasiveness and metastasis of multiple cancers by downregulating pathways driven by the oncoprotein, BMI-1 [27, 29, 33]. BMI-1 induces epithelial-mesenchymal transition (EMT), invasiveness, and metastasis of human nasopharyngeal epithelial cells and GC cells; conversely, BMI-1 silencing reduces cell motility and inhibits EMT [35, 36]. EMT is a mechanism that enables tumor cells to gain mesenchymal cell properties, including higher cell motility and lower cell adhesion, which enables them to metastasize efficiently [37, 38]. Downregulation of E-cadherin and upregulation of vimentin and snail proteins are hallmarks of EMT [39]. In this study, we show that USP22 knockdown significantly decreases migration and invasiveness of GC cells. Moreover, lung metastasis is significantly reduced when USP22-silenced GC cells are injected into the SCID mice via the tail vein. These data suggest that USP22 promotes GC progression.

USP22 and c-Myc are co-expressed in GC tissues [7]. Increased levels of c-Myc in the human colorectal cancer cells positively correlate with high SIRT1 protein expression; furthermore, conditional activation of c-Myc increases levels of SIRT1 protein, NAD^+^, and NAMPT mRNA in several cell types [17]. c-Myc induces the expression of NAMPT, the rate-limiting enzyme of the NAD^+^ salvage pathway, which produces NAD^+^, the essential co-factor required for SIRT1 activity [17]. SIRT1 is a downstream effector of the c-Myc and is activated in several cancers [14, 17]. High SIRT1 levels are associated with tumor progression and prognostic prediction in GC patients [40, 41]. High SIRT1 levels promote EMT, anoikis resistance, and invasiveness of GC cells [42]. SIRT1 also regulates cell survival, senescence, inflammation, and metabolism via deacetylation of histones, p53, NF-κB, and members of the FOXO family [43]. SIRT1 reduces FOXO3a- and FOXO1-induced apoptotic cell death, but promotes induction of cell cycle arrest [19, 44]. SIRT1 repression increases cellular levels of acetylated FOXO1 that transcriptionally activates apoptotic signaling in the lung cancer cells [45]. FOXO1 represses GC growth and angiogenesis; inactivation of FOXO1 is associated with SIRT1 expression in human GC tissues and xenograft GC tumor tissues [46]. In this study, SIRT1 knockdown by siSIRT1 or treatment of GC cells with the SIRT1 inhibitor, EX527, resulted in the upregulation of FOXO1 and pro-apoptotic Bax, and downregulation of cell cycle promoting factors, Ki67 and Cyclin D1, and anti-apoptotic Bcl-2. Previous studies have shown that SIRT1 is a key positive regulator of metastasis that induces EMT in several types of cancers [15]. Moreover, SIRT1-mediated deacetylation regulates YAP in cancer cells [47]. Li *et al.* reported that the SIRT1/YAP signaling pathway promotes the migration of GC cells [20]. YAP1 promotes breast cancer metastasis via EMT [48]. High YAP1 levels are positively associated with lymph node metastasis and poor prognosis of GC [49]. Moreover, ectopic expression of YAP1 induces a more invasive phenotype and accelerates GC cell growth, both *in vitro* and *in vivo* [50]. YAP1 overexpression increases the expression of Vimentin and decreases the expression of E-cadherin in the GC cells [51]. YAP activation also enhances the expression of MMP-2 and MMP-9 in the GC cells [24]. We demonstrate that SIRT1 knockdown using siSIRT1 or inhibition of SIRT1 activity by EX527 treatment significantly reduces the expression of Vimentin, MMP-2 and MMP-9, and increases E-cadherin expression in the GC cells. Overall, these results indicate that USP22 promotes GC progression via c-Myc/NAMPT/SIRT1-dependent FOXO1 and YAP signaling.

Our study has several limitations. The mechanistic details regarding how USP22 promotes proliferation and survival in GC cells requires further in depth study. The role of BMI-1 relative to USP22 in regulating EMT and GC progression is still not known. Moreover, the role of epigenetic mechanisms, transcription factors, noncoding RNAs, post-translational modifications, and other mechanisms required for USP22 expression in GC cells needs to be studied in greater detail.

In conclusion, our data shows that high expression of USP22 positively correlates with GC growth and metastasis. We demonstrate that USP22 is an independent prognostic predictor in GC patients. Our study shows that USP22 regulates the growth and progression of GC cells via c-Myc/NAMPT/SIRT1-dependent FOXO1 and YAP signaling pathways. Our data also suggests that USP22 is a potential prognostic biomarker and a promising target for GC therapy.

## MATERIALS AND METHODS

### Patient information and gastric cancer tissue samples

We studied 266 formalin-fixed paraffin embedded tissue specimens from 186 GC patients that did not undergo any preoperative chemotherapy or radiotherapy before surgery at the 150^th^ Central Hospital of PLA between January 2004 and December 2008. These included noncancerous mucosa (n = 40), primary carcinoma (n = 186), and lymph nodal metastasis (n = 40) tissue samples from the GC patients. The forty noncancerous and lymph nodal metastatic tissues were matching samples obtained from 40 patients diagnosed with primary GC. We also obtained fresh 40 matched GC and adjacent noncancerous tissue specimens for RNA extraction experiments from patients undergoing surgery without receiving chemotherapy or radiotherapy. These samples were immediately frozen in liquid nitrogen and stored at −80°C. Clinical staging of the gastric tumors was performed according to the guidelines proposed in the 7^th^ edition of the American Joint Committee on Cancer. The patients were also followed up until December 2013 or until death, if it happened before this date. The clinical characteristics are summarized in Table 1. We obtained written and signed informed consent from all patients. This study was approved by the Ethics Committee of the 150^th^ Central Hospital of PLA (IRB approval number: CHPIRB-MED-14-321).

### Cell culture

The human GC cell lines, BGC-823, MGC-803, SGC-7901, MKN-45, AGS, and HGC-27, and the normal gastric mucosal epithelial cell line, GES-1, were purchased from the American Type Culture Collection (ATCC; South San Francisco, CA, USA). They were cultured in RPMI-1640 medium (Gibco BRL, Grand Island, NY, USA) supplemented with 10% fetal bovine serum (FBS; Gibco, NY, USA) and 1% penicillin-streptomycin (Sigma–Aldrich, St. Louis, MO, USA) in a humidified chamber maintained at 37°C and 5% CO_2_. The cells from 2-4 passages were used for various experiments.

### Establishment of BGC-823-luciferase cell line

We obtained the pLV-luc lentiviruses from Inovogen Biotechnology (Delhi, India) and infected the BGC-823 cells. We selected stably expressing BGC-823-luc cell line by growing them for 16 days in selection medium containing 200 μg/ml puromycin (Sigma, St. Louis, MO, USA). Representative images of the control and luciferase-expressing BGC-823-luc cells are shown in Supplementary Figure 3.

### Cell transfection and infection

Small interfering RNAs (siRNAs) against USP22 (siUSP22-1 and siUSP22-2), c-Myc (sic-Myc), NAMPT (siNAMPT), SIRT1 (siSIRT1), and negative control (siNC) were purchased from GenePharma (Shanghai, China). The sequences of these siRNAs are: siUSP22-1, 5′-CACGGACAGUCUCAACAAUTT-3′; siUSP22-2, 5′-GGAGAAAGAUCACCUCGAATT-3′; sic-Myc, 5′- GAATTTCTATCACCAGCAA-3′; siNAMPT, 5′-CCA CCCAACACAAGCAAAGUUUAUU-3′; and siSIRT1, 5′-CCCUGUAAAGCU UUCAGAATT-3′. Transfections were performed using Lipofectamine 2000 (Invitrogen, Carlsbad, CA, USA) according to the manufacturer’s instructions.

We obtained lentiviruses with pLKO.1-shUSP22 and pLKO.1 vector constructs from GenePharma to generate stably USP22 depleted and control GC cell lines. The sense and antisense shUSP22 sequences were 5′-AACTC ACGGACAGTCTCAACAA TTTCAAGA GAATTGTTGAGACTGTCCGTGTTTTTC-3′ and antisense 5′-TTGAGT GCCTGTCAGAGTTGTTAAA GTTCTCTTAACAACTCTGACAGGCACAAAAAAG AGCT-3′, respectively. All the constructs were confirmed by DNA sequencing. We obtained stably expressing single clones of pLKO.1- and pLKO.1-shUSP22-transfected BGC823 and HGC-27 cells by selecting for 12 days in growth media containing 200 μg/ml puromycin (Sigma, St. Louis, MO, USA).

### Quantitative real-time PCR assay

Total RNA was isolated from fresh GC and control tissues and cell lines using the Trizol reagent (Invitrogen) in accordance with the manufacturer’s protocol. The cDNA were generated using the Reverse Transcription Kit (TaKaRa, Otsu, Shiga, Japan). Quantitative PCR assay was performed using the SYBR Green PCR master mix (Applied Biosystems, Foster City, CA, USA) in an ABI 7500 Real Time System (Applied Biosystems). The qPCR primers were: USP22, 5′-GACCAGATCTTCACAG GCGG-3′ (forward) and 5′-GCAGACTTGGCAGGTG ACGT-3′' (reverse); GAPDH, 5′-TGAAGGTCGGAGTC AACGG-3′ (forward) and 5′-CTGGAAGATGGTGAT GGGA TT-3′ (reverse). GAPDH was used as the internal control. The relative USP22 gene expression was calculated in different samples using the 2^−ΔΔCT^ method.

### Chromatin immunoprecipitation assay

We cross-linked the control, Myc-silenced, or NAMPT-silenced BGC823 and HGC-27 cells by treating with formaldehyde and then the cells were harvested for the ChIP assay. Then, the chromatin was fragmented by sonication, and pre-cleared. The pre-cleared chromatin was immunoprecipitated with monoclonal antibodies against c-Myc, NAMPT (both from Abcam, Cambridge, UK), or the corresponding IgG isotype control (Abcam, Cambridge, UK) overnight. PCR analysis was performed using primers flanking the NAMPT or SIRT1 promoter region.

### Western blot analysis

The fresh control and GC tissues and cell lines were lysed. The protein lysate was centrifuged. Protein samples were separated by SDS-PAGE, transferred onto PVDF membranes (EMD Millipore, USA). The membranes were then blocked with 5% skimmed milk in Tris-phosphate buffer containing 0.05% Tween 20 (1X TBST) for 1 h at room temperature. Then, after washing with 1X TBST buffer thrice, the membranes were incubated with primary antibodies against USP22, c-Myc, NAMPT, SIRT1 (all from Cell Signaling Technology), FOXO1, Ki67, Cyclin D1, Bax, Bcl-2, YAP, E-cadherin, vimentin, MMP-2, MMP-9 (all from Abcam), and β-actin (Sigma), overnight at 4°C. Then, after washing with 1X TBST buffer, the membranes were incubated with horseradish peroxidase- conjugated secondary antibody (Sigma) for 1 h at 37°C. Then, the blots were developed using an enhanced chemiluminescence kit (Santa Cruz, Dallas, TX, USA) and the protein bands were visualized and quantified.

### MTT cell viability assay

Cell viability was determined using 3-(4,5-dimethylthiazol-2-yl)-2, 5-diphenyl- tetrazolium (MTT; Sigma). GC cells at a density of 1 × 10^3^ cells/well were seeded in 96-well plates, transfected with siUSP22-1 or siNC, and then incubated at 37°C for 1, 2, 3, and 4 days. At each time point, each well was incubated for 4 h after adding 20 μl of 5 mg/ml MTT solution. Then, the culture medium was removed and 150 μl dimethyl sulfoxide (DMSO; Sigma) was added to each well. The optical density (OD) values were measured at 570 nm using a microplate reader (Bio-Tek Instruments Inc., Winooski, VT, USA). Average values were calculated and the growth curves were constructed.

### Colony formation assay

The lentivirus-infected (BGC823-pLKO.1, BGC823-pLKO.1-shUSP22; HGC27- pLKO.1, HGC27-pLKO.1-shUSP22) and mock cells were seeded into 6-well plates at a density of 2 × 10^3^ cells/well. The culture medium was changed every 3 days for 2 weeks. Then, the colonies were fixed by incubating with methanol and stained with 0.5% Crystal violet (Sigma). Images were captured using the Olympus microscope (Tokyo, Japan) and the number of colonies in each sample were quantified and plotted.

### EdU cell proliferation assay

We then determined the role of USP22 in cell proliferation using the EdU (5-ethynyl-2′-deoxyuridine) incorporation assay as described previously [6]. Briefly, USP22-silenced and control GC cells were seeded in 6-well plates and grown for24 h. Then, the cells were incubated with 50 μM EdU (RiboBio, Guangzhou, China) for 2 h and stained with Apollo^®^567 according to the manufacturer’s instructions. After washing thrice with cold phosphate-buffed saline (PBS), cells were incubated with the DNA staining dye Hoechst 33342 (Sigma) for 10 min. The stained cells were photographed using an inverted fluorescent microscope (Olympus) and six random fields were selected to estimate the total number of EdU-positive cells in each of the samples.

### Cell cycle analysis

Two days after transfecting GC cells with siNC or siUSP22-1, the cells were washed thrice with PBS and fixed with cold 75% ethanol at 4°C overnight. Then, the cells were treated with 1 mg/ml RNase and 50 μg/ml propidium iodide (PI; Sigma) in PBS containing 1 μg/ml Triton X-100 at 37°C for 30 min in the dark. The cells were analyzed using the FACSCalibur flow cytometer (BD Biosciences, San Jose, CA, USA). The proportion of cells in the G1, S, and G2/M phases in each sample were calculated using the ModFit software (Verity Software House Inc., Topsham, ME, USA). Each experiment was repeated thrice and the average of triplicate samples was compared to analyze differences between samples.

### Flow cytometry analysis of GC cell apoptosis

Cellular apoptosis in USP22-silenced and control GC cells was determined as previously described using the Annexin V-fluorescein isothiocyanate (FITC)/PI apoptosis detection kit (BD Biosciences) as per the manufacturer’s instructions [6]. Briefly, the GC cells were transfected with siNC or siUSP22-1 for 48 h. Then, the cells were harvested and centrifuged. Single cell suspensions in binding buffer at a density of 1 × 10^3^ cells/ml were incubated with 10 μl Annexin V-FITC at 37°C for 15 min, followed by incubation with 5 μl PI for 30 min in the dark. Then, the stained cells were analyzed using the FACSCalibur flow cytometer (BD Biosciences, San Jose, CA, USA) and the percentage of Annexin-V^+^ PI^+^, Annexin-V^+^ PI^-^, and Annexin-V^-^ PI^-^ cells in each sample were estimated using the CellQuest software (BD Biosciences). Each experiment was analyzed in triplicate.

### Caspase-3 activity assay

Caspase-3 activity was measured using the Caspase-3/CPP32 Colorimetric Assay Kit (BioVision, Palo Alto, CA, USA) according to the manufacturer’s instructions as described previously [6]. We incubated USP22-silenced or control GC cells (1 × 10^6^ per sample) with 50 μl chilled lysis buffer for 10 min. The cell supernatants were collected after centrifugation at 10,000 x*g* for 5 min. Then, 150 μg protein lysates were incubated with 50 μl of 2X reaction buffer containing 5 μl of N-Acetyl-Asp-Glu-Val-Asp-pNA substrate (final concentration: 200 μM) at 37°C for 2 h. The pNA levels that correspond to cleavage of N-Acetyl-Asp-Glu-Val-Asp-pNA by caspase 3 were estimated at 405 nm using a microplate reader (Bio-Tek instruments Inc.).

### Transwell migration and invasion assays

We performed the cell migration assay using 8-μm pore size Transwell inserts (Costar, Dallas, TX, USA). USP22-silenced and control GC cells in RPMI-1640 medium were seeded into the upper chamber of the transwell (2 × 10^4^ cells/insert), and RPMI-1640 medium with 15% FBS was added into the lower chamber. After 24 h, the cells in the lower chamber were fixed in methanol and stained with 4ʹ, 6-diamidino-2-phenylindole (DAPI; Sigma). The total number of cells that migrated was estimated using a fluorescence microscope. The cell invasion assay was similar as the migration assay, except that the bottom of upper chamber was pre-coated with 0.5 mg/ml Matrigel (BD Biosciences). The cells passing through the Matrigel and in the lower chamber were stained with DAPI and estimated using a fluorescence microscope (Olympus). Each experiment was repeated in triplicate.

### Terminal transferase-mediated dUTP nick end labeling (TUNEL) assay

*In situ* DNA strand breaks were detected using the One Step TUNEL Apoptosis Assay Kit (Roche, Mannheim, BW, Germany) according to the manufacturer’s protocols described previously [6]. Briefly, 48 h after siNC or siUSP22-1 transfection, GC cells were seeded onto glass slides and fixed with 80% glycerol at room temperature for 60 min. Then, the cells were rinsed with PBS (pH 7.4), and permeabilized with 0.1% Triton X-100 in PBS. The permeabilized cells were then incubated with FITC-labeled terminal deoxynucleotidyl transferase (TdT) nucleotide mix at 37°C for 60 min in the dark. Then, the cells were rinsed twice with PBS and counterstained with 10 mg/ml DAPI. In tissues, the specimen were fixed with 4% formalin overnight, Then, the fixed tissues were embedded in paraffin, cut into 4 μm thick sections and mounted onto polylysine-covered slides. After incubation in xylene to remove paraffin, the tissue sections were rehydrated by incubation in a graded series of ethanol solutions. Then, the tissue sections were rinsed with PBS, incubated with FITC-labeled TdT nucleotide mix at 37°C for 60 min in the dark, and counterstained with 10 mg/ml DAPI. FITC-labeled TdT was omitted in the nucleotide mix of the negative control. The percentage of TUNEL-positive cells was estimated in all samples (cells and tissues) using fluorescence microscopy (Carl Zeiss).

### IHC assay

IHC assay was performed as previously described to determine USP22 levels in the GC and the corresponding noncancerous tissue samples [6]. The sections were deparaffinized in xylene and rehydrated by incubating in a graded series of ethanol. After heat-induced epitope retrieval, the sections were incubated with 0.3% H_2_O_2_ in methanol for 15 min to quench endogenous peroxidase activity. Then, the sections were incubated with primary antibody against USP22 (1:150 dilution, Cell Signaling Technology) at 4°C overnight followed by incubation for 20 min with the biotinylated secondary antibody using the ChemMate EnVision Kit (DAKO, Hamburg, Germany). The sections were developed by incubation with diaminobenzidine (DAB; Maixin Biotech., Fuzhou, China) and counterstained with hematoxylin (Sigma). For the negative controls, primary antibody was omitted. Blinded analysis of DAB-positive staining was done by two experienced pathologists for all stained specimens. The sections received a score between 0 and 4 according to the percentage of positively stained tumor cells: 0, 0%; 1, 1% to 30%; 2, 31% to 60%; and 3, > 60%. Stain intensity was scored as: 0, negative staining; 1, weak staining; 2, moderate staining; and 3, strong staining. The final staining score (EI) was determined using the formula, EI = *E* × *I*, where ‘E’ refers to score based on percent positively stained cells and ‘I’ refers to staining intensity. The tumor tissues were divided into two categories based on the final staining scores: low-level USP22 group (with a score ≤ 3) and high-level USP22 group (with a score > 3).

### *In vivo* Xenograft and metastasis models

Six-week-old male SCID mice were purchased from the Institute of Zoology, Chinese Academy of Sciences, Beijing, China, and housed under specific pathogen-free conditions. The Ethics Committee of 150^th^ Central Hospital of PLA approved all the animal experimental procedures (IRB approval number: CHPIRB-EA-16-108).

For the xenograft GC tumor growth model, we subcutaneously injected 2 × 10^6^ BGC-823-luc cells containing shUSP22 or the control vector into the hind flanks (*n* = 5 mice/group). Tumor size was measured by a slide caliper every week, and the tumor volume was calculated as (length × width^2^)/2. Tumor growth was monitored by *in vivo* luciferase activity imaging of the xenografts at day 21 after injection. We injected D-luciferin (Promega, Madison, WI, USA) intraperitoneally into mice from both groups at a dose of 150 mg/kg. The animals were then anesthetized and photographed using the Xenogen IVIS imaging system. The luciferase activity signals in the defined regions of interest were quantified as photon flux (photons/s/cm^2^) using the Living Image software (Xenogen Corporation, Berkeley, CA, USA). All the mice were sacrificed at 6 weeks. The tumors were removed and photographed. Tumor samples were fixed, paraffin embedded, and cut into thin sections to be used for performing the TUNEL assay.

For *in vivo* analysis of the effects of USP22 on GC metastasis, SCID mice were injected with 1 × 10^7^ BGC-823-luc cells containing stably-expressed shUSP22 or the control vector (*n* = 5 mice/group) through the tail vein. The mice were sacrificed at four weeks after injection of the GC cells, and their lungs were harvested and photographed. The lungs were then fixed in 4% formalin, embedded in paraffin and thin sliced sections were cut. The lung sections were stained with hematoxylin and eosin (Sigma). The sections were observed under a light microscope (Olympus), and the total number of metastases per lung section was counted for each of the mice. The average or mean numbers of metastases for each group of mice were compared to determine differences.

### Statistical analysis

Statistical analyses were performed using the SPSS 17.0 software (SPSS, Chicago, IL, USA). The experimental values were presented as mean ± standard deviation (SD). The differences between groups were determined by the Student’s t test or one-way analysis of variance. The relationships between the levels of USP22 expression and the clinicopathological features were evaluated using the Chi-square test. Kaplan-Meier survival curve analyses and log-rank tests were used to determine survival differences between patients with high and low USP22 expression. A *p* value of < 0.05 was considered statistically significant.

## Supplementary Material

Supplementary Figures
