# Supplementary material for: Oncogenic USP22 supports gastric cancer growth and metastasis by activating c-Myc/NAMPT/SIRT1-dependent FOXO1 and YAP signaling
Source: Aging (Albany NY). 2019 Nov 4;11(21):9643–60. doi: 10.18632/aging.102410 (PMC6874452; doi:10.18632/aging.102410)
Supplement: Supplementary Figures [file aging-11-102410-s001.pdf]

SUPPLEMENTARY FIGURES

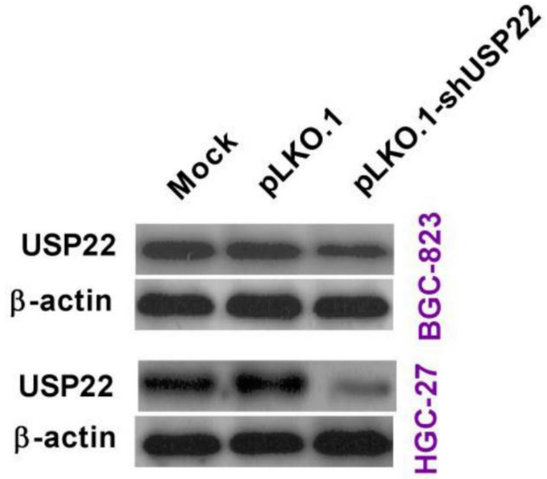

Supplementary Figure 1. The inferring efficiency of pLKO.1-shUSP22 in GC cells. BGC-823 and HGC-27 cells were uninfected (Mock) or infected with lentiviruses pLKO.1 or pLKO.1-shUSP22. The knockdown efficiency of shUSP22 was analyzed by Western blot assay at 24 h after infection. Representative results of western blot analyses of USP22. β-actin was used as endogenous control.

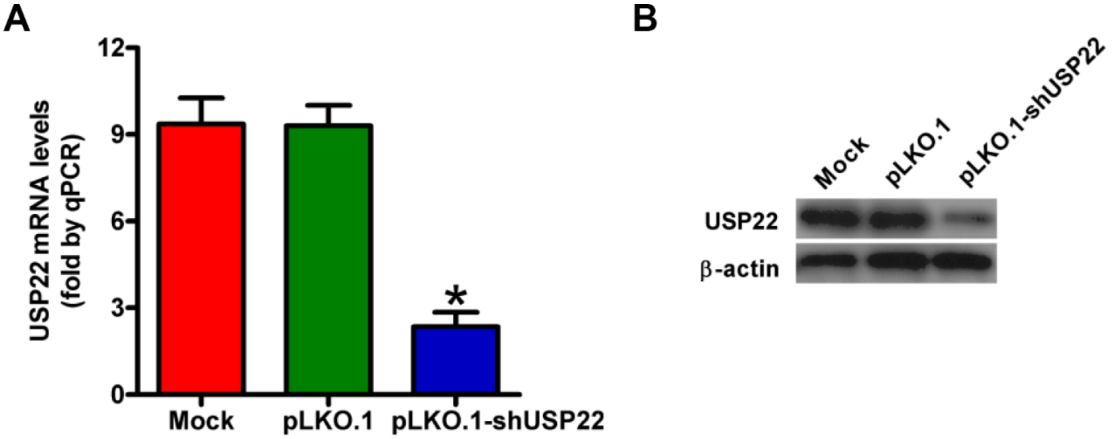

Supplementary Figure 2. mRNA and Protein levels of USP22 in GC tissues from pLKO.1 or pLKO.1-shUSP22 groups. Female six-week-old SCID mice were inoculated subcutaneously into right hind flanks or injected via tail vein with stably expressed pLKO.1 or pLKO.1-shUSP22 BGC-823-luc cells. Mock-treatment was used as control. The mice were sacrificed 6 weeks after tumor cell implantation. (**A**) qPCR assay was performed to detect the mRNA expression of USP22 in tumor tissues. GAPDH was used as endogenous control. (**B**) Protein expression of USP22 in tumor tissues was analyzed by western blot. β-actin was used as endogenous control. The data were from three independent experiments. Bar graph represented mean ± SD. $^*P < 0.05$ compared with mock or pLKO.1 group.

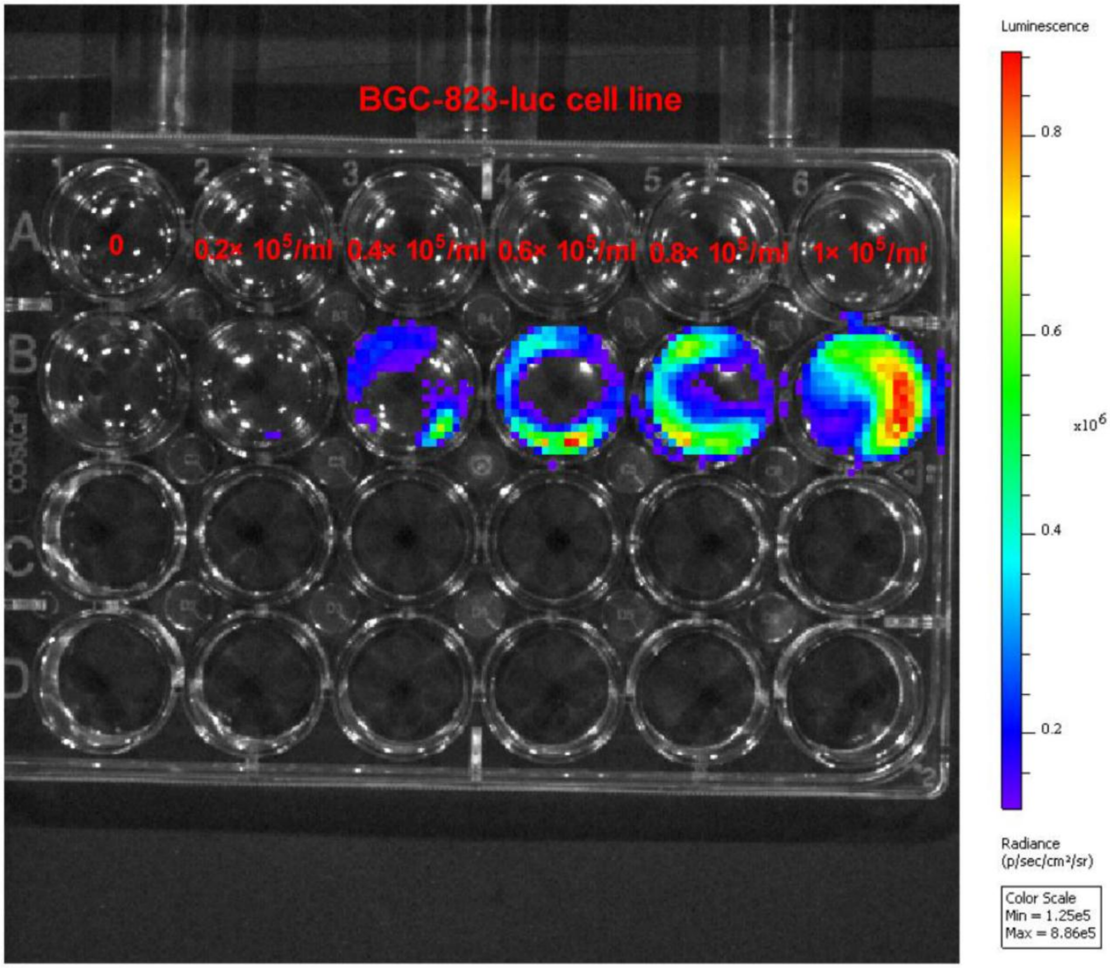

Supplementary Figure 3. The bioluminescence data along with the representative images of BGC-823-luc cells. The different concentrations (0, 0.2×10^5/ml, 0.4×10^5/ml, 0.6×10^5/ml, 0.8×10^5/ml, and 1×10^5/ml) of BGC-823-luc cells were seeded in a 24-well plate. After 48h of culture, the images were acquired using the Xenogen IVIS imaging system. The signals in defined regions of interest were quantified as photon flux (photons/s/cm2) using Living Image software (Xenogen Corporation, Berkeley, CA, USA).
